# On Elixirs Containing Iron

**Published:** 1871-08

**Authors:** W. W. Seay


*On Elixirs Containing Iron.* By W. W. Seay.

A considerable discussion has been carried on regarding the propriety of furnishing the various elixirs for the use of the profession and the public. My own experience has suggested a decided impression in their favor, and, while they can never take the place of similar officinal preparations, they can be made, with proper care, fully equal in medicinal effect, and certainly more agreeable to the taste. Whilst deprecating any fundamental change of our old time-honored and tried remedies, I think many of them could be improved in flavor and appearance, and do our art no discredit. I have known patients absolutely refuse to take many of our tonics long before their use should have been discontinued, simply on the ground of their nauseous, bitter taste. These elixirs are elegant in appearance, and have a decidedly pleasant flavor, which I think is a great consideration where medicines have to be taken for a length of time. As furnished us by large manufacturing firms, my observations are opposed to their usefulness, since in nearly every instance they lack the necessary strength. Certain salts of iron can be added to almost any of our tinctures (modified somewhat in preparation) without precipitation, and prove as useful as before their addition. I propose furnishing a few recipes, the formulæ of which I have originated and found useful. I do not claim any great scientific achievement for them, but I do think they are preferable to any I have yet seen in use. They retain their *tannic acid and natural combinations apparently unchanged, at all events, without any great chemical disturbance.* It will be observed that alcohol of officinal dilute strength is taken, and the drugs exhausted by it, and *then* sugar is added, which *increases the bulk* without interfering with its *solvent action.* The general full dose being in most cases about one-half fluid ounce, the spirituous strength will amount to only one and one-third fluid drachms of officinal alcohol, which quantity cannot be considered very objectionable. Wines or brandies can be substituted for a portion of alcohol, if deemed advisable, account being taken of the difference of alcoholic strength, the object being to approach as nearly as possible to the menstruum ordered for similar officinal tinctures. Like all tinctures, they are directed to be taken in a little water. The subjoined preparation of Elixir Cinchonæ et Ferri Hypophosphitis, I can specially recommend as an elegant and beautiful one, having taken it myself for some time past, where phosphorus was indicated. About its permanence beyond a few weeks I cannot vouch, as I have not laid by any samples for a great length of time to test it. Hypophosphorous acid has a great affinity for oxygen, and how far the sugar will protect it in the presence of organic matter I am unable to state. I have kept it on hand without change for about two months. The solutions can be kept separately and mixed as wanted, and an elegant preparation insured.

*Elixir Cinchonæ et Ferri Hypophosphitis.*

| | |
|---|---|
| Elixir Cinchonæ, - - - - - - - - - - | Oj. |
| Syr. Ferri Hypophosphitis, - - - - - - - - - | f℥ij. |
| Alcoholis Fortior., - - - - - - - - - - | fʒss. |
| Ac. Hypophosphorosi, - - - - - - - - - | f℥iv. |

Mix the syrup, acid and alcohol together, and then add to elixir.

The Elixir Cinchonæ designated is that published by me in the June number Am. Journal of Pharmacy, 1871. The Syr. Hypophosphite Iron is that of W. S. Thompson, and the Hypophosphorous Acid that of Parrish, in Parrish's excellent "Treatise on Pharmacy."

The dose for an adult would be about one-half fluid ounce, containing thirteen grains red Peruvian bark, nearly one-half fluid drachm of the Syrup of the Ferrous Hypophosphite, six and one-half minims of ten per cent. hypophosphorous acid.

*Elixir Gentianæ et Ferri Chloridi.*

| | |
|---|---|
| Gentian, in coarse powder, - - - - | two troy ounces. |
| Recent Orange Peel, bruised, - - - - | two troy ounces. |
| Cardamom Seed, powdered, - - - - | one-half troy ounce. |
| Alcohol Dil., q. s. | |

Percolate s. a. until twenty-one fluid ounces have been obtained. To this add and dissolve

| | |
|---|---|
| Sacch. Alb. Pulv., - - - - - - | thirty-one av. ounces. |
| Acid. Muriat. Pur., - - - - - - | f℥ij. |
| Sol. Ferri Chloridi ($FeCl$), - - - - - | f℥iv. |

The strength of the elixir is about the same as officinal tinctura gentianæ co.; it contains the equivalent of ten drops officinal tr. ferri chloridi to each fluid ounce.

Any elixir that may be desired with chloride of iron can be made in the same way, substituting other drugs instead of gentian. I will furnish my recipes with other salts of iron, for future numbers of the Journal, as I can find the time. I think if druggists will give these recipes a fair trial, they will find the resulting elixirs will give better results than any that have heretofore been published.—*Am. Jour. Pharmacy.*

